# P-76. Age-Dependent Diagnostic Yield and Treatment in Pediatric Bone and Joint Infections

**DOI:** 10.1093/ofid/ofaf695.305

**Published:** 2026-01-11

**Authors:** Alec Wesolowski, Saul I Favela, Joel Rose-Kamprath, Rachel Downey, Marisol Fernandez

**Affiliations:** Dell Children's Medical Center, Austin, TX; University of Texas at Austin Dell Medical School, Austin, Texas; The University of Texas at Austin Dell Medical School, Austin, TX; Dell Children's Medical Center of Central Texas, Austin, Texas; Dell Children's Medical Center of Central Texas, Dell Medical School at UT Austin, Austin, Texas

## Abstract

**Background:**

Molecular testing of surgical specimens is recommended to increase the yield of bacterial identification in culture negative cases of pediatric bone and joint infections (BJI); particularly in pre-school aged patients < 5 years at risk for *Kingella kingae*. The clinical benefit of polymerase chain reaction (PCR) for BJI in children > 5 years, however, remains less clear. This study evaluates the diagnostic yield and added clinical value of PCR testing for BJI in patients < 5 and ≥5 years old at a tertiary pediatric center.

**Table 1.**
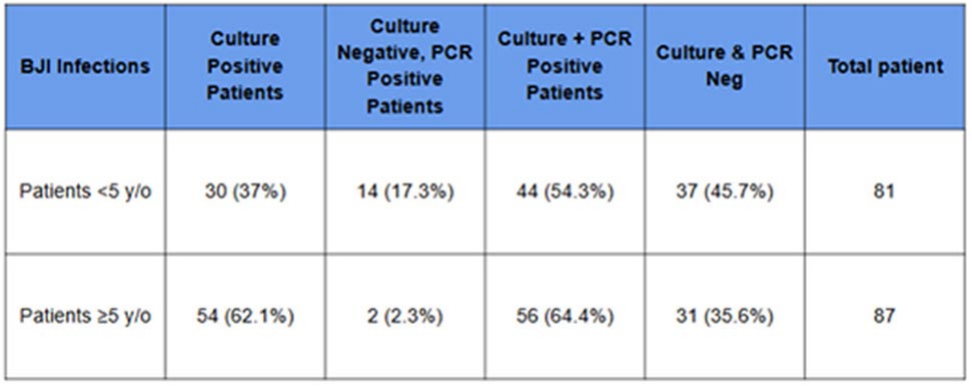
Diagnostic Yield of BJI via Culture and PCR

**Table 2.**
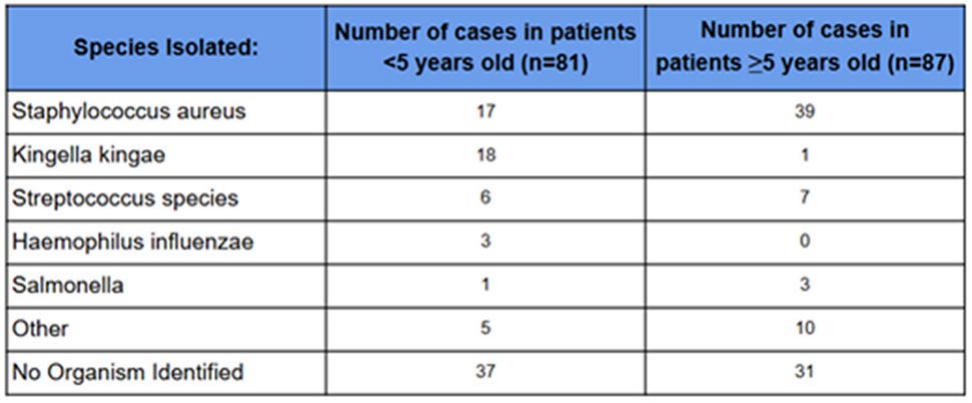
Isolated Organisms in BJI via Culture and PCR

**Methods:**

This single-center, multi-site, retrospective cohort study included pediatric patients admitted between January 2015 to April 2024 in Central Texas diagnosed with BJI. Cases were reviewed for demographics, diagnosis of BJI, antimicrobial treatment, results of blood, bone and synovial fluid cultures, results of PCR testing, and readmission related to BJI treatment failure. Cases were compared by age (< 5 years vs > 5 years) for bacterial identification and benefit of PCR testing. Oral antibiotics at discharge were assessed to determine if positive culture or molecular testing impacted antimicrobial choice.

**Figure 1.**
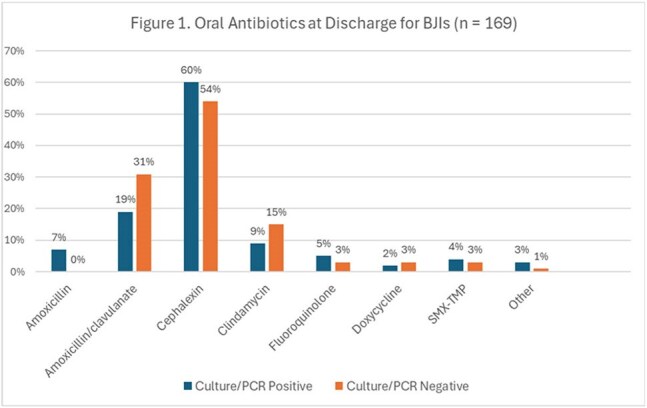
Oral Antibiotics at Discharge for BJIs

**Results:**

168 patients were included (81 patients < 5 years, 87 patients ≥5 years). Cultures were positive in 37% patients < 5 years and 62.1% patients ≥5 years. Molecular testing increased organism identification to 54.3% and 64.4%, respectively. *Staphylococcus aureus* and *K. kingae* were the most common pathogens identified. Amoxicillin/clavulanate or cephalexin was prescribed at discharge in a majority of patients regardless of organism identification. Amoxicillin/clavulanate was prescribed more often in patients < 5 years with negative cultures due to concern for *K. kingae*. One case with confirmed methicillin-susceptible *S. aureus* treated with cephalexin required readmission for treatment failure.

**Conclusion:**

The incorporation of PCR significantly enhanced diagnostic yield in patients < 5 years with BJI, particularly through detection of *K. kingae.* However, regardless of pathogen identification, a majority of patients were discharged on either amoxicillin/clavulanate or cephalexin. Further research is warranted to assess the clinical benefit of PCR testing in pediatric BJI, where its impact on clinical outcomes remains uncertain.

**Disclosures:**

All Authors: No reported disclosures

